# The ‘Lost’ frontier of clinical medicine: Have we reached a point of no return?

**DOI:** 10.4103/0974-2069.52801

**Published:** 2009

**Authors:** Bharat Dalvi

Last 3 decades have brought in technical progress and newer skills that have dramatically improved outcomes in congenital heart disease. There is no denying that the state-of-art imaging and other diagnostics contribute immensely to accuracy of diagnosis, which, of course, is the key to planning a successful therapeutic strategy. Likewise, on the therapeutic front, transcatheter interventions have made possible the safe and facile correction of a variety of congenital heart defects. Major advances in intensive care and surgical techniques have substantially reduced perioperative morbidity and mortality. Unfortunately, the glamour of these successes has shifted the focus away from clinical cardiology which now is relegated to a mundane background.

Just to exemplify the extent of this neglect of clinical medicine, here are two real life case scenarios. The first, was a 4 year old boy admitted with pneumonia and a leukemoid reaction. The high white cell count (85,000/cu mm) was investigated on the fast track with bone marrow examination and esoteric WBC markers, computed tomography (CT) scan of the thorax and abdomen. The stage was set for a mediastinal lymph node biopsy and he was sent to look at his heart on echo before the procedure. He had a perimembranous ventricular septal defect (VSD) with tricuspid valve endocarditis presenting as pneumonia. No one had bothered to listen to the VSD murmur!

The second case was that of an 18-year-old immigrant girl of Asian origin, who came to the emergency room with acute onset breathlessness and X-ray chest showing bilateral fluffy shadows over the lung fields. She was treated for *Pneumocystis carinii* pneumonia and also extensively investigated for acquired immunodeficiency syndrome as she had a history of being previously treated for tuberculosis. Because of non-improvement of ventilatory parameters she was subjected to a two-dimensional echo, which revealed a tight mitral stenosis! One can understand the problem of hearing a low-frequency murmur in the presence of pulmonary edema and rapid heart rate. But, the harsh apical thrill was completely overlooked!

These cases represent a significant cohort of missed diagnoses due to inadequate clinical evaluation, overemphasis on investigations and misinterpretation of test results. In both cases, the correct diagnosis was ultimately reached but at a significant cost in terms of time, morbidity and expense. It also emphasizes the fact that there are no shortcuts in medicine and unless one goes through the systematic drill of inspection, palpation, percussion and auscultation, one is likely to go astray. Investigations at best can supplement but not substitute clinical examination.

It is important to realise that a detailed clinical assessment is critical to deciding the nature and order of investigations. For example, it is not unusual to see an 8 year-old child with neurocardiogenic syncope being subjected to a variety of biochemical tests, ECG, Holter monitoring, echocardiography, CT scan, magnetic resonance angiography, EEG and whatever else technology offers! All test results are expected to be normal. In such a case, what could clinch the diagnosis is a good history, which is so very typical. Defensive medicine has become the present day “mantra.” One of the arguments put forth by those who believe in ordering a battery of investigations is to “play safe.” This is partly due to ready access to diagnostics and partly a fear of litigation. Pressure from the industry and the hospital administrations looking to boost profits have also fuelled the increase in the number of tests advised. Detailed history taking and a good physical examination do not add to the bottom line – neither for hospitals nor the health care industry. They may actually do the reverse and therefore are left as orphans with no takers!

In the early 80s, while doing my residency in internal medicine, I distinctly remember presenting a case of atrial septal defect to the consultant on rounds. Just as I finished presenting the auscultatory findings, he turned and asked “Have you ruled out myasthenia gravis in this boy?” I was stumped. Within the short time that I narrated the history and physical findings, he had observed an asymmetry in the palpebral fissures due to mild ptosis on one side – that was his reason to suspect myasthenia! As I went into the details of history and examined him more thoroughly, he did turn out to have myasthenia gravis. He had an underlying thymoma, which was successfully operated upon. Twenty-five years ago, there was no ultrasound, CT scan or magnetic resonance imaging in Mumbai. I am not sure how long it would have taken to diagnose that thymoma had it not been for his astute clinical judgement. This case is an illustration of what human senses and a sharp mind can do, and importantly that machines cannot replace man.

A number of studies have consistently shown that the incidence of wrong diagnoses has not changed significantly despite the availability of modern diagnostic modalities.[1] Echocardiography, ultrasound and CT scans are known to mislead the physicians as much if not more than the clinical history and physical examination.[2] This is to be expected because every investigation has its share of false-positives and false-negatives. Investigations need to be interpreted in the context of a clinical setting. Only too often, we tend to treat the report rather than the patient.

Over the past couple of decades, practice of clinical medicine has been deteriorating gradually.[3] Although this has been a global phenomenon, it has taken a greater toll in the developing world with resource constraints where clinical evaluation has a significant role to play in decision-making for patient management. One of the main reasons for the declining standards of clinical medicine is the lack of good and dedicated teachers.[4] Gone are the days when teachers taught for the love of teaching. That breed is almost on the verge of extinction. Teaching is no more a sought-after profession because the financial compensation given to the teaching staff in the public sector medical schools is woefully inadequate when compared with their counterparts in clinical practice. Some others opt for private practice to get away from the unwanted interference and politicization by administrators and bureaucrats. Most of the smart minds are being recruited by the rapidly growing private sector health care facilities, leaving the next generations of medical students at the mercy of a handful of good teachers. Unless governments and educational bodies take solid measures on a war footing to retain talent in the teaching faculty, I think we will continue our journey southwards. To add to this problem, we now have coaching classes trying to tutor students in clinical skills in the classrooms with the help of simulators, typical case scenarios and other computer-based modules. These surrogate teaching modalities cannot replace bedside patient interaction supervised by a dedicated and passionate teacher.[5] This reminds me of Sir William Osler who said, “He who studies medicine without books sails an uncharted sea, but he who studies medicine without patients does not go to sea at all.”

The impact of this dire state of clinical medicine may not become apparent for a few more years because we still have a large pool of clinicians trained by master teachers. However, if we wait to see the repercussions of a population treated by doctors with inadequate training in clinical medicine, it may be too late and the implications too grave. The writing on the wall is very clear. Thus, we must act; act effectively and act now.
